# C6-ceramide treatment inhibits the proangiogenic activity of multiple myeloma exosomes via the miR-29b/Akt pathway

**DOI:** 10.1186/s12967-020-02468-9

**Published:** 2020-08-03

**Authors:** Liping Liu, Qinmao Ye, Langni Liu, Ji Chen Bihl, Yanfang Chen, Jing Liu, Qian Cheng

**Affiliations:** 1 Department of General Surgery, The Third Xiangya Hospital, Central South University, Changsha, 410013 Hunan China; 2 Department of Physiology and Cell Biology, The Dorothy M. Davis Heart and Lung Research Institute, The Ohio State University, Columbus, OH USA; 3 Department of Pharmacology and Toxicology, Boonshoft School of Medicine, Wright State University, Dayton, 45435 USA; 4 Department of Hematology, The Third Xiangya Hospital, Central South University, Changsha, 410013 Hunan China

**Keywords:** Multiple myeloma, Exosomes, C6-ceramide, MiR-29b, Akt pathway

## Abstract

**Background:**

The increased bone marrow angiogenesis is involved in the progression of multiple myeloma (MM) with the underlying mechanism poorly understood. Cancer-released exosomes could play an important role in the pathological angiogenesis through exosomal microRNAs (miRs) delivery. It is reported that miR-29b played an important role in regulating the tumor angiogenesis.

**Methods:**

In this study, we explored the role of C6-ceramide (C6-cer, a Ceramide pathway activator) in the angiogenic effect of MM exosomes and its potential mechanism. MM cells (OPM2 and RPMI-8226) treated with C6-cer were studied for its effects on the endothelial cell (EC) functions.

**Results:**

Our results showed that exosomes released from MM cells treated by C6-cer (Exo^C6-cer^) significantly inhibited the proliferation, migration and tube formation of ECs. For mechanism studies, we found that the level of miR-29b was increased in ECs treated by Exo^C6-cer^, while mRNA and protein expressions of Akt3, PI3K and VEGFA were decreased in ECs, indicating the involvement of Akt pathway. Furthermore, downregulation of miR-29b by inhibitor administration could prevent the Exo^C6-cer^-induced cell proliferation, migration and angiogenesis of ECs, accompanied with the increased expressions of Akt3, PI3K and VEGFA.

**Conclusions:**

Collectively, our data suggest that Exo^C6-cer^-mediated miR-29b expression participates in the progression of MM through suppressing the proliferation, migration and angiogenesis of ECs by targeting Akt signal pathway.

## Background

Multiple myeloma (MM) is a kind of clonal B cell neoplasm that accounts for more than 10% of all hematologic malignancies [1]. Over the past years, the applications of autologous stem-cell transplantation and chemotherapeutic agents such as lenalidomide, and bortezomib have improved the overall survival. Despite these therapeutic options available, the disease remains incurable and the standard survival is up to 4 years. Increased angiogenesis is a constant hallmark of MM progressions and is crucial for its growth, invasion and metastasis [2, 3]. VEGF-A, one of the important members in the family of vascular endothelial growth factor (VEGF), can induce angiogenesis, promote MM cell proliferation and migration [4]. In recent years, angiogenesis has become one of the most important targets in therapies used in oncology and the anti-angiogenic therapies have proven itself to be very promising in treatment of MM.

During MM development, it is reported that MM cells can affect endothelial cells through cell–cell contact or secretion of soluble factors to build up a favorable microenvironment [5]. Emerging evidence suggest that extracellular vesicles shed from MM cells act as an essential mediator of crosstalk in the Bone Marrow(BM) [6, 7]. Exosomes are 30–150 nm membrane vesicles that derived from various cancer cells and are involved in modulating cell to cell communications [8]. Biogenesis of exosomes initiates as an important event in its functions [8]. It has been reported that ceramide pathway were involved in exosomes biogenesis and secretion [9]. Ceramide is one of the central molecules of sphingolipid metabolism and plays a critical role in the regulation of various cellular functions, including cell proliferation, migration and apoptosis [9]. Some studies reported that ceramide contributes to tumor suppressive and anti-proliferative cellular functions [10–12]. We previously found that the ceramide pathway could modulate the proliferation, apoptosis and exosome functions of MM [13]. However, whether this pathway could also affect the communications between MM cells and endothelial cells remains elusive. C6 ceramide (C6-cer) is a short-chain, cell-permeable analog of ceramide that has been utilized in preclinical studies to mimic the effects of endogenous ceramide [12]. We have previously showed that C6-cer treated MM exosomes could inhibit the proliferation of MM cells, indicating the anti-tumor role of C6-cer by exosomes [13]. However, whether C6-cer treated MM cell-derived exosomes could affect angiogenesis remains largely unknown.

MicroRNAs (miRs), a kind of small, non-coding RNAs, are considered to be important components of cancer signaling network and are emerging as novel biomarkers of disease [14, 15]. Exosomal miRs play an essential role in controlling cell proliferation, apoptosis and migration of targeted cells. It has been found that miR-29b is highly associated with the proliferation and survival of MM cells [15]. C6-cer has been shown to increase the exosomal levels of some tumor-suppressive miRs (miR-29b, miR-202, and miR-15a/16) [13]. Furthermore, it has been shown that miR-29b can be used as high effective anti-cancer therapeutic agent by simultaneously exert the effects including both anti-angiogenesis and anti-tumorigenesis via regulating the Akt pathway [16]. In spite of all the findings on miR-29b with respect to the involvement in MM, the specific mechanism of exosomal miR-29b in anti-angiogenesis of MM has not been explicitly elucidated.

In this study, we investigated whether targeting the ceramide pathway by using the C6-cer could exert the anti-angiogenesis in MM. Moreover, the role of exosomal miR-29b and its potential targets will also be investigated.

## Materials and methods

### Cell culture

MM cells (OPM2 and RPMI-8226 cell line) were generously gifted by Dr. Zhan (University of Iowa). Cells were cultured in RPMI-1640 medium (Hyclone, USA) with 10% fetal bovine serum (FBS) (Hyclone, USA) and maintained at 37  ℃ with 5% CO_2_ and 95% humidity. C6-cer (Sigma, USA) was routinely stored at − 80 °C as a 5 mM stock suspension in DMSO. Right before use, the suspension was solubilized by the addition of PBS and diluted into work solution. The suspension was completely mixed and warmed up at 37 °C until clear.

### Exosome isolation, detection and quantification

*Collection* Exosomes were prepared from culture supernatants of MM cells treated by 10 µM C6-cer (Exo^C6-cer^) by differential centrifugations. Briefly, cells were maintained in their recommended media until they reach 70–80% confluency. Then, the cell medium was collected and centrifuged at 300 g for 15 min, followed by centrifugation at 2000 g for 30 min to remove cells and cell debris. The cell-free culture medium was centrifuged at 20,000 g for 70 min and then ultracentrifuged at 170,000 g for 1.5 h to pellet exosomes. The pelleted exosomes were resuspended with 20 nm filtered (Whatman, PA) phosphate-buffered saline (PBS).

*Particle size and concentration analysis* The analyses of size and concentration were in consistent with our previous studies [13, 14]. In details, exosomes were resuspended in PBS, the particle size and concentration were measured by using the Nano Tracking System 300 (NTA300, Malvern Instruments, UK). In this study, diluted suspensions containing exosomes were loaded into the sample chamber, and the camera level was maintained at 10 for light scatter mode and at 16 for fluorescence scatter mode between samples. Light scatter mode of the tracking system used the camera filter 1. Three videos of typically 30 s duration were taken, with a frame rate of 30 frames per second. Data was analyzed by NTA 3.0 software (Malvern Instruments) which was optimized to first identify and then track each particle on a frame-by-frame basis.

### Cell proliferation assay

Cell proliferation was determined by the 3-(4, 5-dimethylthiazol-2-yl) -2, 5-diphenyltetrazolium bromide (MTT) assay. HUVECs were seeded in 96-well culture plates (Thermo Fisher Scientific) at a concentration of 4 × 10^3^ cells per well. After incubation with Exo^C6-cer^ (40ug/ml) for 48 h, 5 μL of 10 mg/mL MTT was added to each well. The plates were further incubated for 4 h at 37 °C and 1 M NaOH solution supplemented with 1% SDS were added to each well for at least 12 h. Absorbance was measured at 490 nm with a microplate spectrophotometer (Bio-Tek, USA).

### Cell migration assay

Cell migration was determined by the wound-healing assay. HUVECs (1 × 10^5^ cells per well) were grown to 80% confluence in a 24-well plate. Wounds were made by scraping a conventional pipette tip across the cell monolayer. Cell migration was induced by DMEM supplemented with 10% FBS. The wound images were captured by microscopy (Olympus, Japan) immediately after wounding and after 12 h. The wound width was measured using the ImageJ software, and the percentage of wound healed was calculated using the following formula:

$${\text{Wounded area filled }}\left( \% \right)\, = \, 100\% \, - ({\text{width after 12}} {\text{h}}/{\text{width at the beginning}})\, \times \, 100\% .$$

### Endothelial cell capillary-like tube formation assay

Matrigel was liquefied at 4 °C and pipetted into precooled 96-well plates (50 μl/well) and polymerized for 30 min at 37 °C. HUVECs treated with Exo^C6-cer^ were paved onto the surface of the Matrigel (2.5 × 10^4^ per well) and observed for 12 h. The tubular networks were quantified by the tube sprouting rates. Tube sprouting rate (%) = (sprouted cells/total cells) × 100.

### Protein extraction and western blot analysis

Whole-cell lysates and total exosomal proteins were prepared by using RIPA buffer (50 mM Tris–HCl pH 7.4, 150 mM NaCl, 1% NP40, 0.5% sodium deoxycholate, 0.1% sodium dodecyl sulfate). 50ug total proteins were electrophoretically separated on 4–12% SDS-acrylamide Gel (Thermo Fisher Scientific). Akt3 and PI3K p110α were examined in the study. Western blot analyses were performed with primary antibodies: anti- β-actin, anti-Akt 3, anti-P-Akt, anti-PI3K, anti-VEGFA (1: 1000, Cell Signaling Technology, USA), and the corresponding secondary antibodies anti-mouse and anti-rabbit peroxidase-linked (1: 10 000; Cell Signaling Technology, USA). The signals were visualized by ECL Prime Western Blotting Detection Reagent (Advansta, USA).

### RNA extraction and quantitative real-time PCR of miRs

Total RNA was isolated using Trizol Reagent (Invitrogen, USA) according to the manufacturer’s instruction. The purified RNA was subjected to reverse transcription reactions by using PrimeScriptTM RT reagent kit (Takara, Japan). QRT-PCR with SYBR Premix Ex Taq II Mix (Takara, Japan) was used to evaluate the expression of the genes and U6 as an internal control. QRT-PCR assays were performed in StepONEPlus Real-Time PCR system (Thermo Fisher Scientific) and the relative expression was calculated using the comparative 2^−ΔΔCt^ method.

### Statistical analysis

Statistical significance was calculated via Microsoft Excel using a Student t-test (one-sided) or ANOVA as appropriate. Data generated displayed normal distribution with similar variances, and analysis was performed assuming equal variances.

## Results

### The characteristics of exosomes derived from MM cells treated with C6-cer

**Fig. 1:**
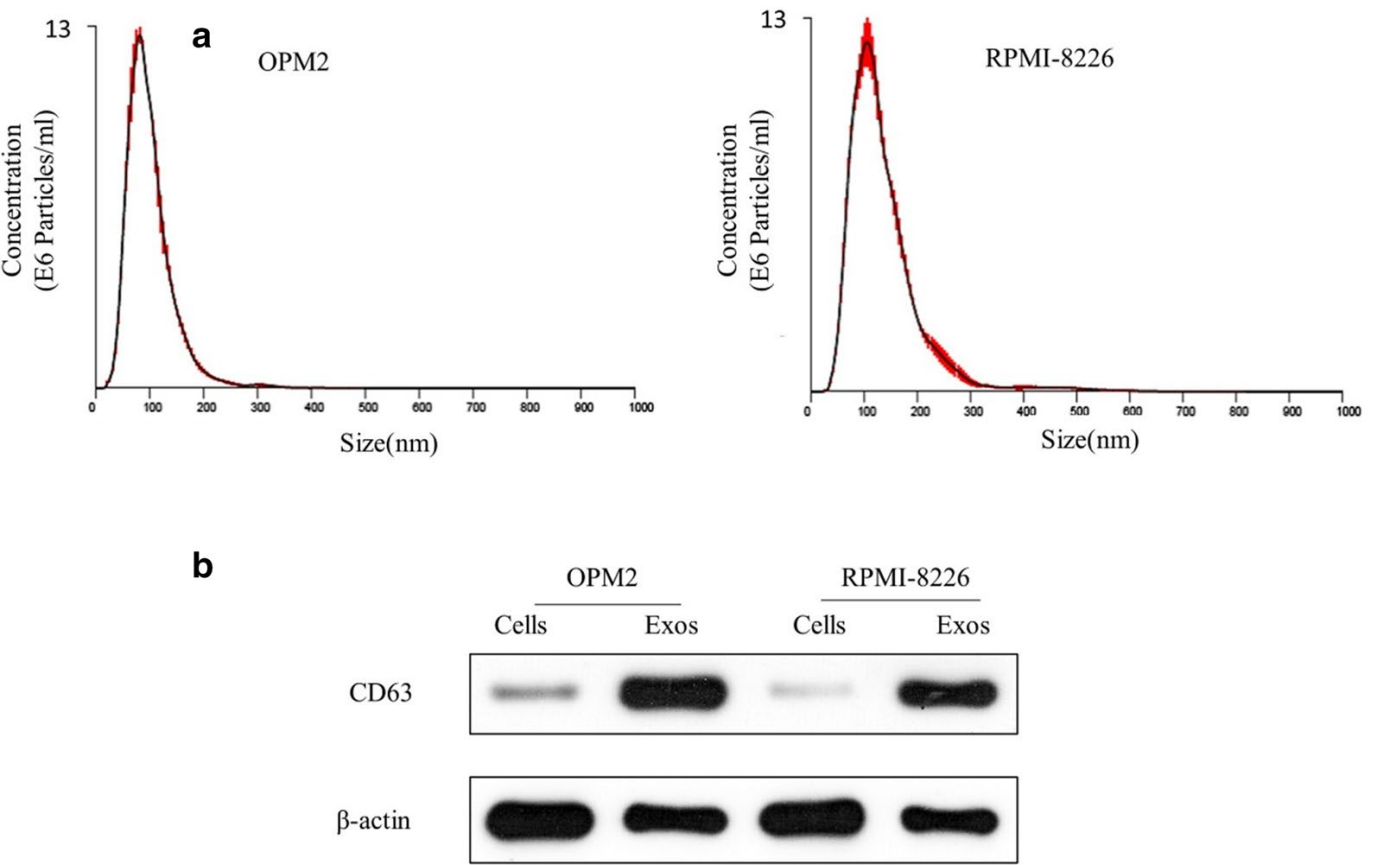
The characteristics of exosomes derived from MM cells treated with C6-cer. **a** Representative plots showing the size/concentration distribution of the secreted exosomes isolated from MM cells (OPM2 and RPMI-8226) treated with C6-cer tested by NTA 300; **b** The western blot analysis was performed to assess the level of CD63, which is the exosomal maker. All the experiments were performed in triplicates

To identify the characteristics (size and concentration), exosomes isolated from MM cells (OPM2 and RPMI-8226) treated with C6-cer (10 µM) were analyzed. NTA 300 revealed that the sizes of isolated exosomes were about 100 nm (Fig. 1a). The western blot analysis was performed to assess the level of CD63, which is the exosomal maker (Fig. 1b). As expected, CD63 was highly enriched in the exosomes compared to the cell lysates, indicating the high purity of exosomes.

### Effects of Exo^C6-cer^ on the functions of HUVECs

**Fig. 2:**
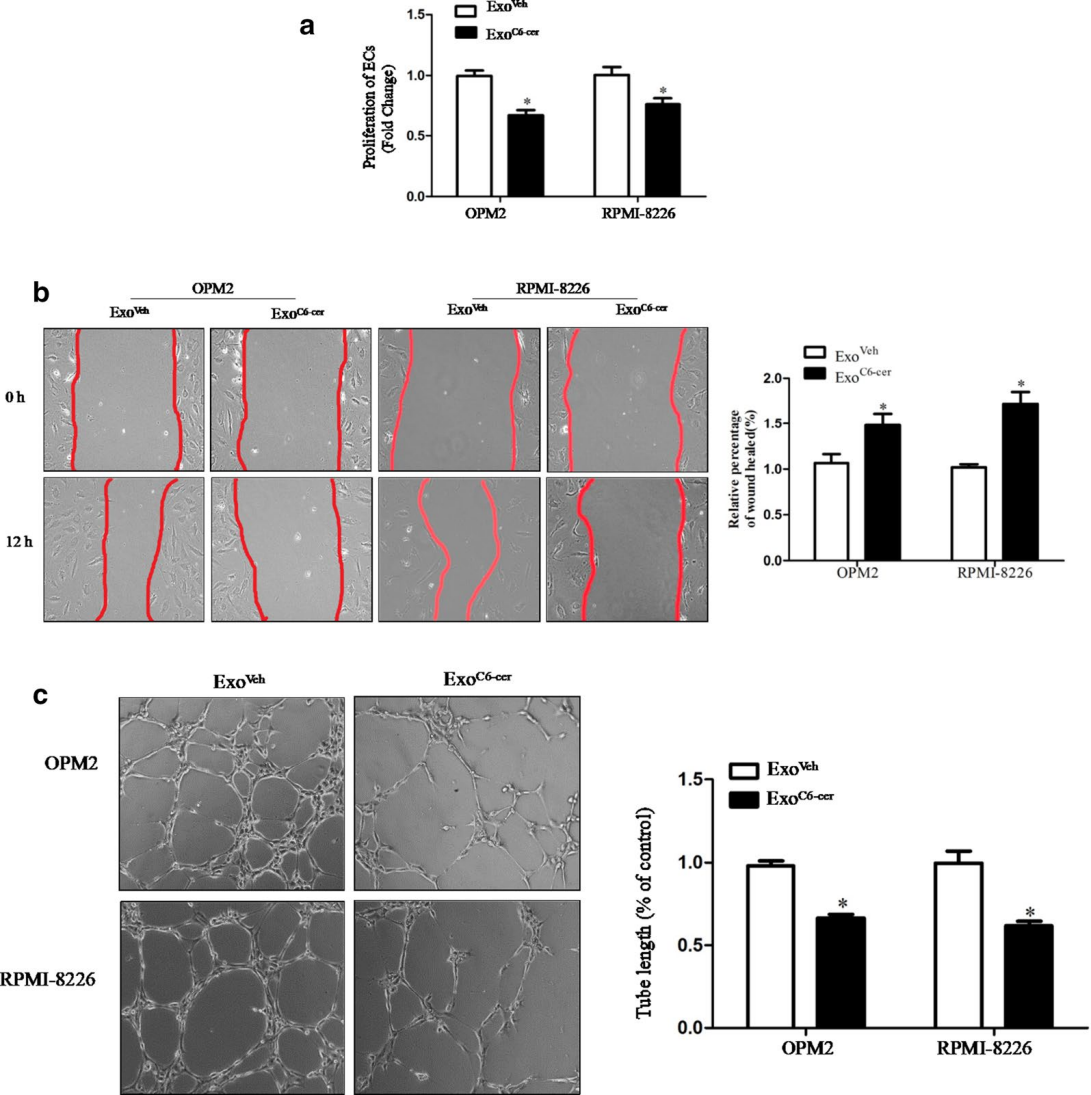
Effects of Exo^C6-cer^ on the functions of HUVECs. **a** The proliferation of HUVECs was tested after 48 h of incubation with 40 µg/ml of Exo^C6-cer^ by MTT assay. **b** Migration assays were performed to explore the effects of Exo^C6-cer^ on the migration abilities of HUVECs, which was photographed under a microscope at × 100 magnification (left-hand panels). The mean wound width was measured and the images are representative of three independent experiments (right-hand panel). **c** Tube formation of HUVECs treated differently was photographed under a microscope at × 100 magnification (left-hand panels). All the experiments were performed in triplicates. Three fields were counted under the microscope. The images are representative of three independent experiments. Mean tube length was quantified by image pro-plus software (right-hand panel). (*P < 0.05)

Next, we investigated the effects of Exo^C6-cer^ on the proliferation, migration and angiogenic activity of HUVECs. As shown in Fig. 2a, a significant increase in cytotoxicity of HUVECs was achieved after 48 h of incubation with 40 µg/ml of Exo^C6-cer^ by MTT assay. On the other hand, the co-culture of Exo^C6-cer^ with HUVECs inhibited the invasive capacity of HUVECs (Fig. 2b). We evaluated the tube formation in HUVECs to determine the effects of Exo^C6-cer^ on the angiogenic activity in vitro. As shown in Fig. 2c, Exo^C6-cer^ significantly inhibited tube formation of HUVECs. The anti-angiogenic activity of Exo^C6-cer^ was shown at a concentration of 40 µg/mL.

### Effects of Exo^C6-cer^ on the expressions of miR-29b and Akt pathway in HUVECs

**Fig. 3:**
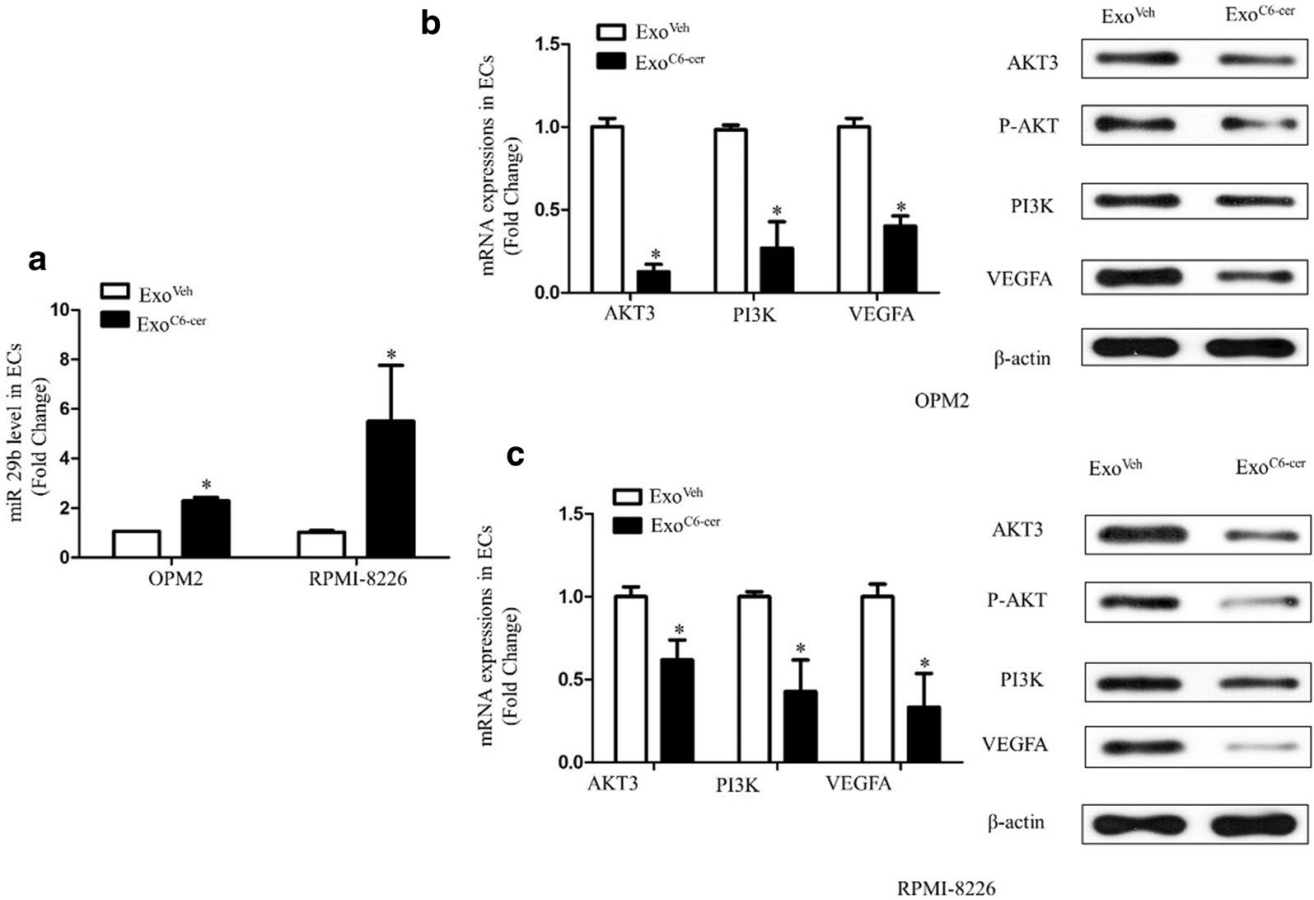
Effects of Exo^C6-cer^ on the expressions of miR-29b and Akt pathway in HUVECs. **a** The expression of miR-29b in HUVECs was quantified by using real-time PCR after co-cultured with Exo^C6-cer^ for 48 h. **b** qPCR showed the expressions of Akt3, PI3K and VEGFA in OPM2 after Exo^C6-cer^ treatment (left-hand panels). Western blots showed the expressions of Akt3, P-AKT, PI3K and VEGFA in OPM2 after Exo^C6-cer^ treatment (right-hand panel). β-Actin is used as normalization control for the total lysate. The data shown were representatives of three independent experiments that gave similar results. **c** qPCR showed the expressions of Akt3, PI3K and VEGFA in RPMI-8226 after Exo^C6-cer^ treatment (left-hand panels). Western blots showed the expressions of Akt3, P-AKT, PI3K and VEGFA in RPMI-8226 after Exo^C6-cer^ treatment (right-hand panel). β-Actin is used as normalization control for the total lysate. All the experiments were performed in triplicates. The data shown were representatives of three independent experiments that gave similar results. (*P < 0.05)

The expression of miR-29b in HUVECs was quantified by using real-time PCR after co-cultured with Exo^C6-cer^. We analyzed the potential targets of miR-29b via bioinformatics and found that the Akt3 might be one of the following targets of miR-29b. The expression of miR-29b in HUVECs was significantly increased after adding Exo^C6-cer^ into HUVECs culture for 48 h (Fig. 3a), indicating that extracellular miRs, derived from C6-cer treated MM cells, transferred into HUVECs. Meanwhile, qPCR and Western Blot results showed that the expressions of Akt3, PI3K and VEGFA were significantly decreased in HUVECs after Exo^C6-cer^ treatment (Fig. 3b, c), indicating the involvement of Akt pathway.

### Inhibition of miR-29b on the functions of HUVECs and the expressions of Akt pathway in HUVECs

**Fig. 4:**
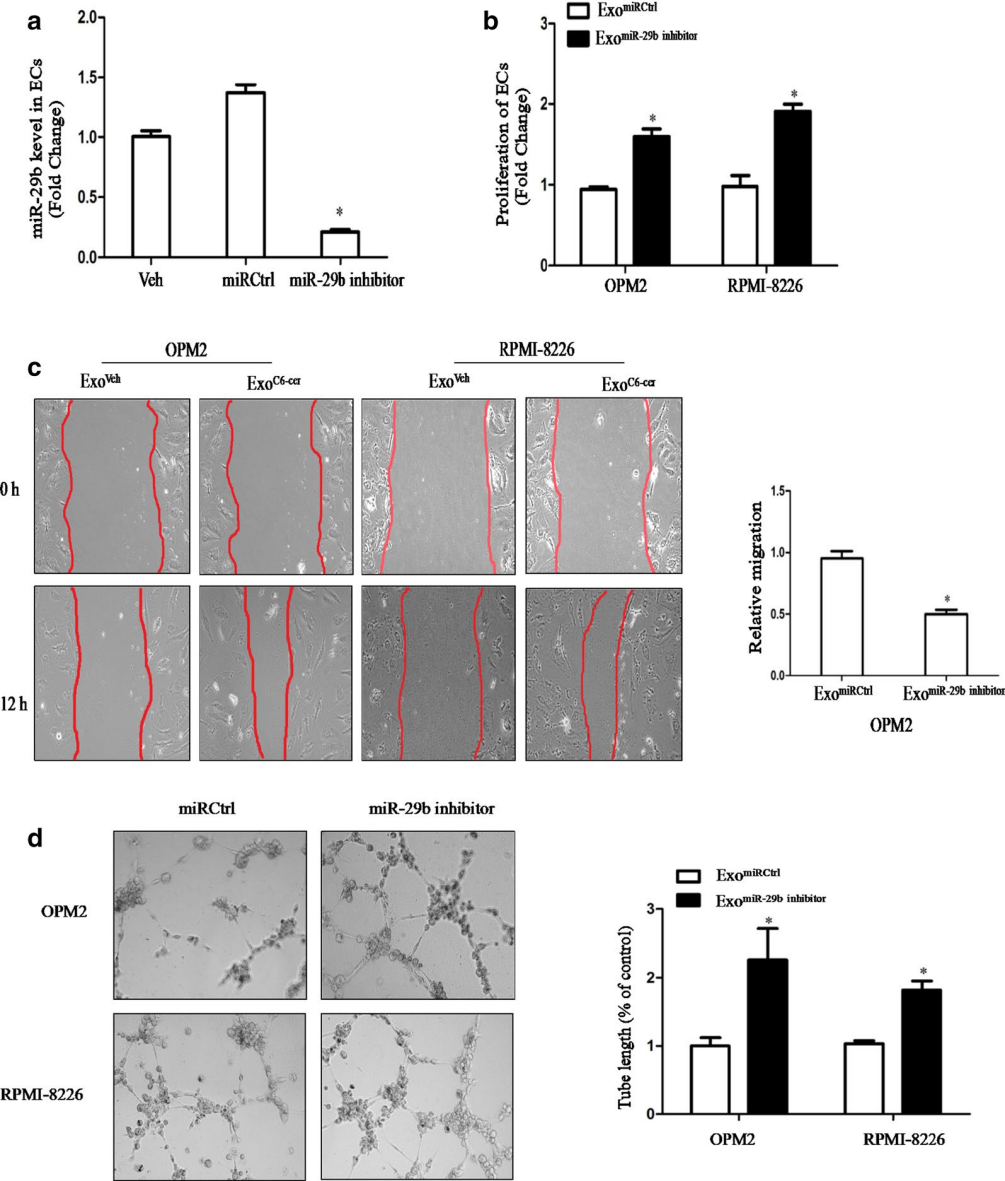
Inhibition of miR-29b on the functions of HUVECs. **a** The expression of miR-29b in HUVECs was tested by qRT-PCR analyses after the transfection of miR-29b inhibitor. **b** The proliferation of HUVECs was tested after 48 h of incubation with 40 µg/ml of Exo^C6-cer^ by MTT assay. **c** Migration assays were performed to explore the effects of Exo^C6-cer^ on the migration abilities of HUVECs, which was photographed under a microscope at × 100 magnification (left-hand panels). The mean wound width was measured (right-hand panel). The images are representative of three independent experiments. **d** Tube formation of HUVECs treated differently was photographed under a microscope at × 100 magnification (left-hand panels). Mean tube length was quantified by image pro-plus software (right-hand panel). All the experiments were performed in triplicates. The images are representative of three independent experiments. (*P < 0.05)

**Fig. 5:**
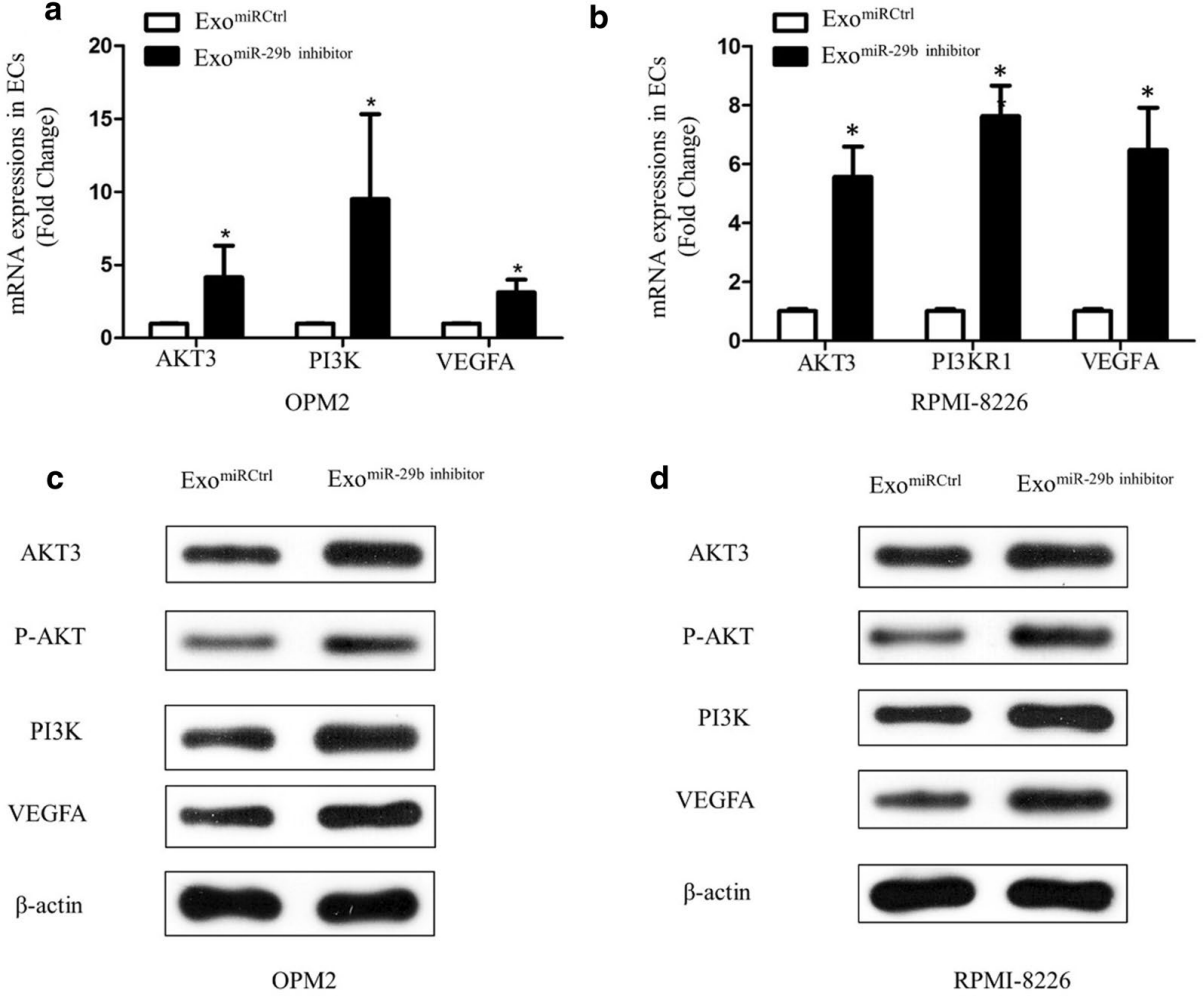
Inhibition of miR-29b on the expressions of Akt pathway in HUVECs. **a** The qPCR analysis showed the expressions of Akt3, PI3K and VEGFA in OPM2 after Exo^miR-29b inhibitor^ treatment. **b** The qPCR showed the expressions of Akt3, PI3K and VEGFA in RPMI-8226 after Exo^miR-29b inhibitor^ treatment. β-Actin is used as no^iR-29b inhibitor^ treatment. **c** Western blots showed the expressions of Akt3, P-AKT, PI3K and VEGFA in OPM2 after Exo^miR-29b inhibitor^ treatment. β-Actin is used as normalization control for the total lysate. **d** Western blots showed the expressions of Akt3, P-AKT, PI3K and VEGFA in RPMI-8226 after Exo^miR-29b inhibitor^ treatment. β-Actin is used as normalization control for the total lysate. The experiments were performed in triplicates. The data shown were representatives of three independent experiments that gave similar results. (*P < 0.05)

To further demonstrate the effect of miR-29b on the expressions of Akt pathway, the miR-29b inhibitor was performed to inhibit the expression of miR-29b in HUVECs. HUVECs were infected with miR-29b inhibitor and then co-cultured with Exo^C6-cer^ (40 µg/ml). The expression of miR-29b in HUVECs was inhibited by 75% after transfection (Fig. 4a). After inhibiting the expression of miR-29b, results showed an increased proliferation (Fig. 4b) and invasive activity (Fig. 4c) in HUVECs. As shown in Fig. 4d, the cumulative tube length was increased in HUVECs treated with miR-29b inhibitor compared to HUVECs treated with miR Ctrl, indicating that low expression of miR-29b enhanced the pro-angiogenic capacity of exosomes. Meanwhile, the qPCR and Western Blot results showed that the expressions of Akt3, PI3K and VEGFA were significantly increased of HUVECs (Fig. 5), indicating the involvement of Akt pathway.

## Discussions

In the present study, we demonstrate a significant inhibition of ECs proliferation, migration and angiogenesis by targeting the ceramide pathway via C6-cer treated MM derived exosomes. Two distinct human myeloma cell lines of OPM2 and RPMI-8226 were utilized in our study. We also observed that the level of miR-29b was increased in C6-cer exosomes treated ECs. Further mechanism study identified Akt pathway might be a direct target of miR-29b in ECs and indicated that miR-29b can reduce EC angiogenesis probably via post-transcriptional inhibition of Akt/PI3K/VEGF expression. These findings also suggest that therapeutic targeting of ceramide and miR-29b/Akt pathway might be a viable alternative strategy to MM.

Ceramide, a bioactive sphingolipid, is thought to induce death, growth inhibition, and senescence in cancer cells [17]. Pro-cell death function of ceramide suggests that ceramide mimics or analogues may open doors to new therapies to battle cancer. Thus, finding ways to increase ceramide either by exogenous treatment or by elevating endogenous ceramide in cancer cells is desired [17]. Moreover, the ceramide pathway is closely associated with the biogenesis of exosomes which turn out to be a novel target of cancer therapies. We previously found that exosomes derived from C6-cer treated MM cells (Exo^C6-cer^) could inhibit the proliferation and increase the apoptosis of MM cells, indicating the therapeutic roles of C6-cer by exosomes [13]. In this study, our results expanded these findings and found that exosomes derived from C6-cer treated MM cells could also inhibit the proliferation, migration and angiogenesis of ECs. Increasing angiogenesis is a favorable avenue for delivering oxygen and nutrition to the growing MM cells. Therefore, controlling this process is a promising therapeutic approach in preventing cancer progression.

MiRs, present an important role between exosomes and their targeted cells. MiRs has been shown the therapeutic promise as clinical candidates in MM. Emerging evidence demonstrated that miR-29b suppresses cell proliferation, migration and angiogenesis in a variety of cancer cells including breast [16], gastric [18] and ovarian cancer [19], indicating the miR-29b might be a critical regulator in tumorigenesis. However, its role in tumor angiogenesis remains underexplored. We found that the miR-29b was significantly increased in Exo^C6-cer^ treated ECs, implicating a high level of miR-29b in Exo^C6-cer^ and was consistent to our previous study [13].

One of the major pro-angiogenic factors is VEGF which has been shown to be a potent stimulator of angiogenesis in vitro [20]. We found that the Exo^C6-cer^ could inhibit the expressions of VEGF probably via targeting the Akt and PI3K. Our results showed that transfection of miR-29b inhibitor successfully upregulated the expression of VEGF, and subsequently promoted angiogenesis by increasing cell proliferation and migration. Mechanistically, we found that miR-29b was involved in the regulation of Akt3/PI3K expressions and thereby modulated the expression of VEGF in HUVECs. Akt3 is known to induce VEGF secretion and angiogenesis in tumor. Akt3 and PI3K were confirmed to be the downstream target genes of miR-29b in ECs. Our data demonstrated that decreased miR-29b expression was accompanied by significantly increased Akt3 and PI3K protein expressions.

C6-ceramide may make a difference in the content of exosome, which could affect its function. Due to the time and space limitation, we did not examine its exact effects in the content of exosome. In our future work, we will perform control experiments to compare and identify the effect from ceramide-induced apoptosis on the exosome secretion. As another limitation, we did not perform animal experiments or human sample investigation. In the future, we will further prove the functions of Exo^C6-cer^ from MM in human MM samples, perform animal experiments, and translate the results of our current study to clinical field.

It was already demonstrated that exosomes derived from MM cells treated with C6-cer could inhibit the proliferation of MM cells. As the uniqueness in our work, we expanded the investigation of C6-ceramide in another area, and revealed the functions of Exo^C6-cer^ on the proliferation, invasion and angiogenesis of HUVECs. Our findings could be a promising therapeutic strategy in biological delivery for MM therapies.

## Conclusions

In this study, we have further discussed the functions of Exo^C6-cer^ from MM. Our data suggest that Exo^C6-cer^-mediated miR-29b expression participates in the progression of MM through suppressing the proliferation, migration and angiogenesis of ECs by targeting Akt signal pathway.

## Supplementary information

**Additional file 1:** Raw figures.

## Data Availability

The data sets supporting the results of this article are included within the article and Additional file 1: Raw figures.
