# Supplementary material for: C6-ceramide treatment inhibits the proangiogenic activity of multiple myeloma exosomes via the miR-29b/Akt pathway
Source: J Transl Med. 2020 Aug 3;18:298. doi: 10.1186/s12967-020-02468-9 (PMC7398185; doi:10.1186/s12967-020-02468-9)
Supplement: Supplementary file 1 — Additional file 1: Raw figures. [file 12967_2020_2468_MOESM1_ESM.pptx]

## Slide 1
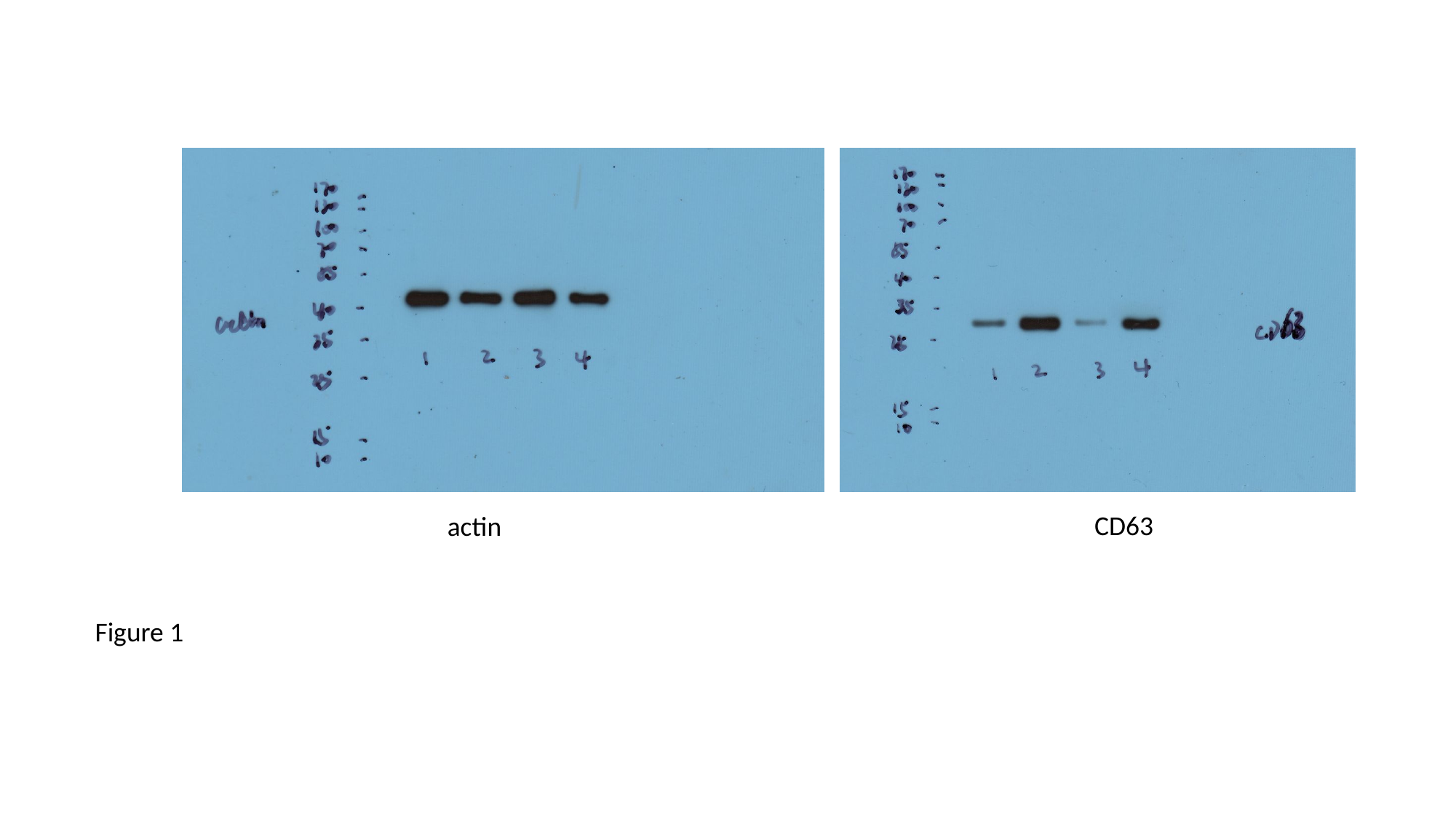

CD63
actin
Figure 1

## Slide 2
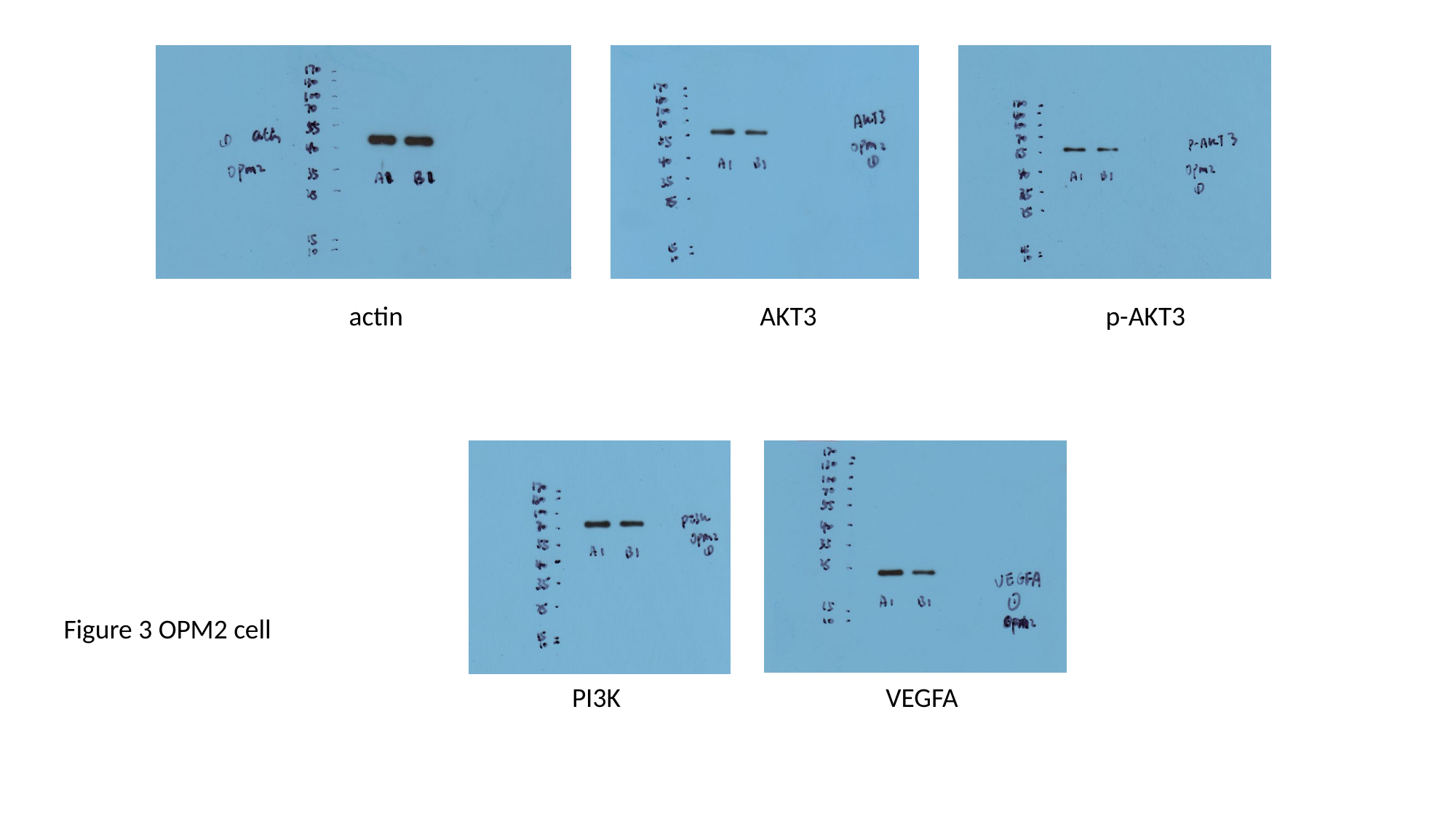

actin
AKT3
p-AKT3
Figure 3 OPM2 cell
PI3K
VEGFA

## Slide 3
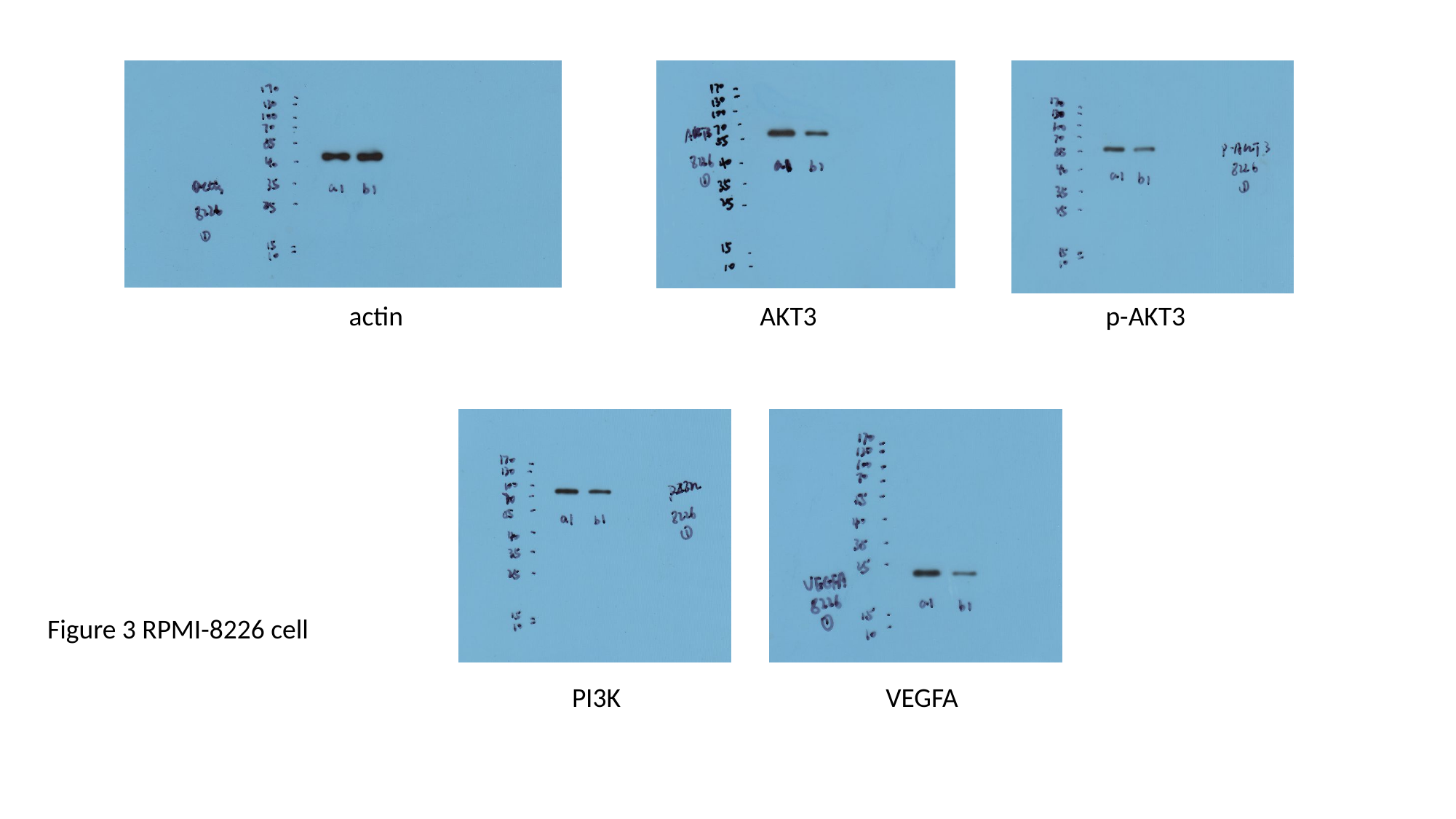

actin
AKT3
p-AKT3
Figure 3 RPMI-8226 cell
PI3K
VEGFA

## Slide 4
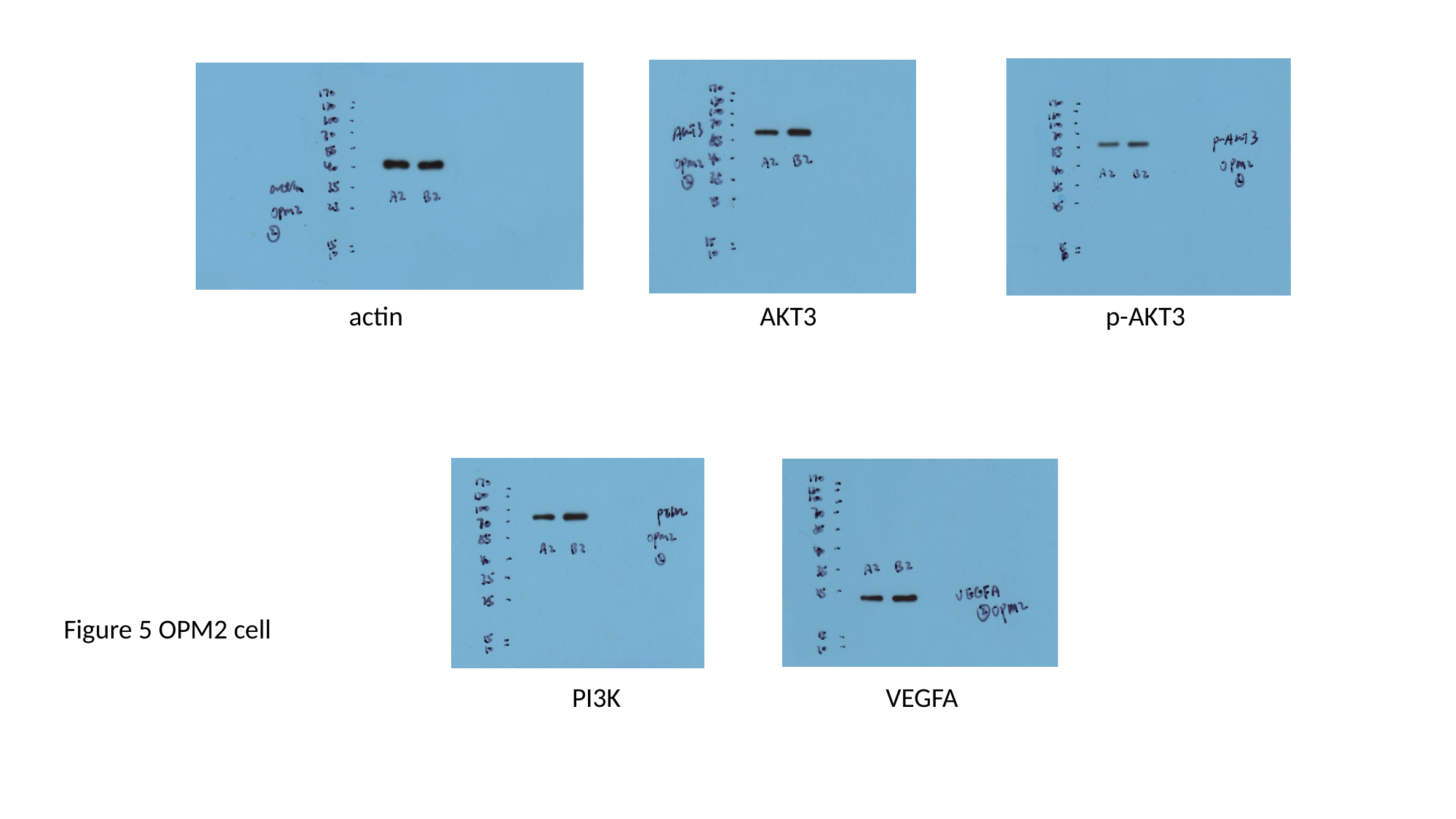

actin
AKT3
p-AKT3
Figure 5 OPM2 cell
PI3K
VEGFA

## Slide 5
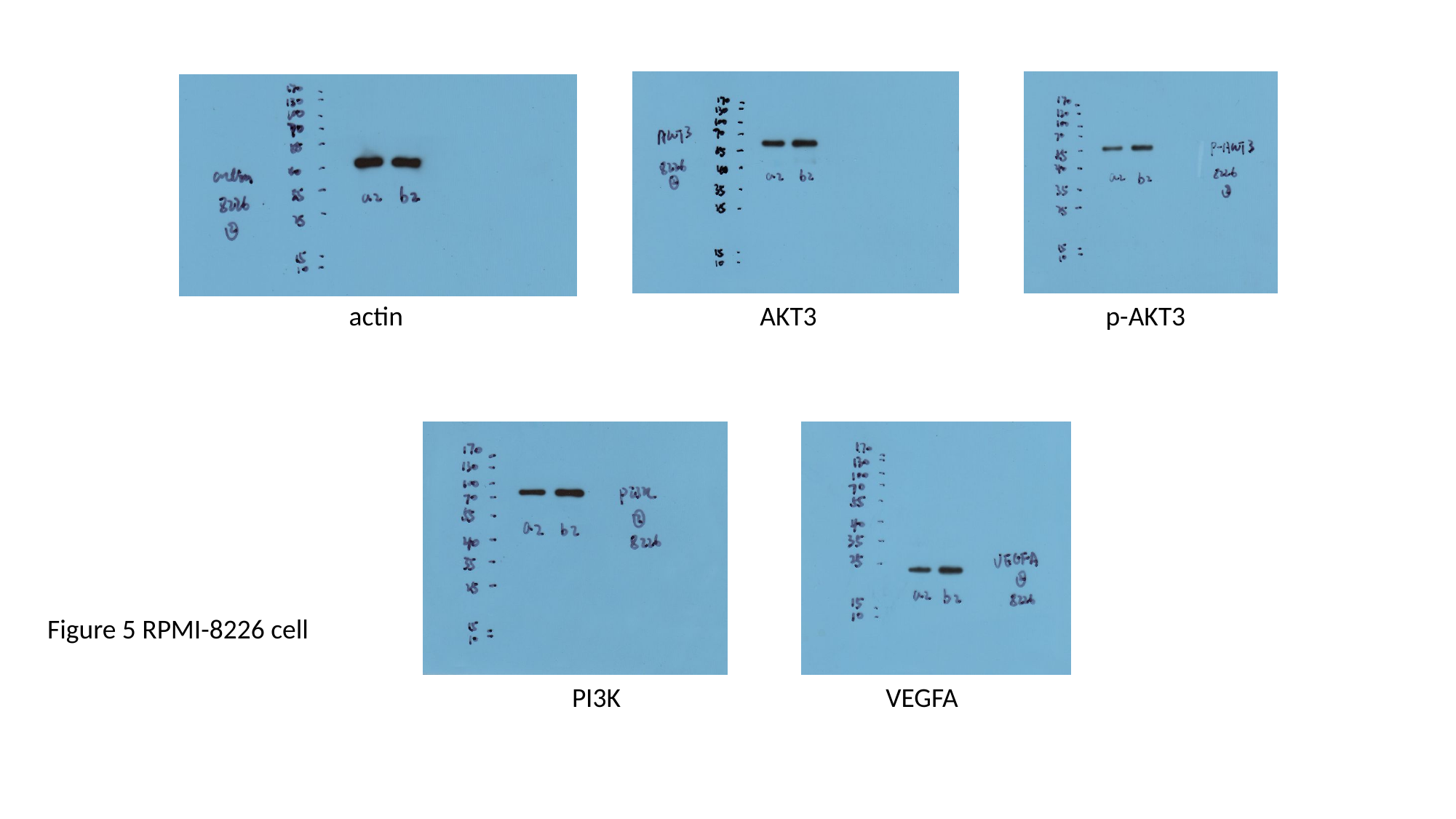

actin
AKT3
p-AKT3
Figure 5 RPMI-8226 cell
PI3K
VEGFA
